# Neutrophilic and eosinophilic dermatoses associated with hematological malignancy

**DOI:** 10.3389/fmed.2023.1324258

**Published:** 2024-01-05

**Authors:** Carlo Alberto Maronese, Federica Derlino, Chiara Moltrasio, Daniele Cattaneo, Alessandra Iurlo, Angelo Valerio Marzano

**Affiliations:** ^1^Dermatology Unit, Fondazione IRCCS Ca’ Granda Ospedale Maggiore Policlinico, Milan, Italy; ^2^Department of Pathophysiology and Transplantation, Università degli Studi di Milano, Milan, Italy; ^3^Hematology Division, Foundation IRCCS Ca’ Granda Ospedale Maggiore Policlinico, Milan, Italy; ^4^Department of Oncology and Hemato-Oncology, University of Milan, Milan, Italy

**Keywords:** neutrophilic, eosinophilic, dermatoses, pyoderma, sweet

## Abstract

Cutaneous manifestations of hematologic malignancy represent both a clinical challenge for the treating physician and a pathophysiological model for advancing the knowledge on individual neoplasms. Indeed, a growing body of evidence supports the concept of recurrent molecular defects associating with specific clinical features, as best exemplified by VEXAS. Herein neutrophilic and eosinophilic dermatoses of potential interest for both hematologists and dermatologists will be reviewed, including subcorneal pustular dermatosis-type IgA pemphigus, neutrophilic eccrine hidradenitis, Sweet’s syndrome as well as myelodysplasia cutis and VEXAS, pyoderma gangrenosum, eosinophilic annular erythema, eosinophilic dermatosis of hematological malignancy, Wells syndrome and cutaneous involvement in hypereosinophilic syndromes. Possible management approaches are discussed for each, emphasizing scenarios that require treatment of the underlying condition to achieve remission at the skin level.

## 1 Introduction

Cutaneous manifestations of hematologic malignancies (HMs) represent both a clinical challenge for the treating physician and a pathophysiological model for advancing the knowledge on individual neoplasms. Indeed, a growing body of evidence supports the concept of recurrent molecular defects associated with specific clinical features, as best exemplified by VEXAS (vacuoles, E1 enzyme, X-linked, autoinflammatory, somatic) (1). Moreover, acquired somatic mutations in the hematopoietic compartment, other than those documented in true HM, have been documented to fuel a minor proportion of autoinflammatory urticaria, providing yet another example of the complex relationship between hemoproliferative disorders and the skin (2, 3).

While different subsets of cutaneous presentations have been linked to hematologic tumors (4), herein only neutrophilic and eosinophilic dermatoses will be reviewed, due to their special interest for both hematologists and dermatologists.

Neutrophilic dermatoses (ND), also known as neutrophilic diseases, are classified according to their clinico-pathological picture into superficial/epidermal, dermal and deep forms, with a fourth category that encompasses mixed as well as syndromic ND (5). Eosinophilic dermatoses have been classified similarly (6), but it should be underscored that available evidence is relatively more limited for this group. Levels of Evidence are also introduced for each of the discussed entities (7).

## 2 Neutrophilic dermatoses

### 2.1 Superficial neutrophilic dermatoses

#### 2.1.1 Subcorneal pustular dermatosis-type IgA pemphigus (level of evidence 4)

IgA pemphigus is a rare neutrophilic acantholytic autoimmune disease, with two subtypes that differ in terms of epidermal immunoglobulin (Ig)A deposition patterns: subcorneal pustular dermatosis (SPD) and intraepidermal neutrophilic IgA dermatosis (IEN) (8).

SPD-type IgA pemphigus is a relapsing, sterile dermatosis clinically characterized by flat, hypopyon pustules, often on a slightly erythematous base. Histologically, it shows subcorneal acantholysis, pustules with intercellular IgA deposits in the upper epidermis and predominant neutrophil infiltration (9). True SPD differs from it for the negativity of immunofluorescence studies (9).

Concomitant lymphoproliferative disorders have been reported in SPD-IgA pemphigus, especially IgA monoclonal gammopathy of uncertain significance (MGUS) with a rate of 9.5% (10). It is speculated that IgA paraproteinemia may affect neutrophil function and migration, however the exact pathogenesis of the relationship with HM is presently unknown.

Cases of IgA multiple myeloma (MM) have also been reported, with six patients described so far (11–15). In four cases, the onset of MM was concomitant with the diagnosis of IgA pemphigus while in the remaining two patients, MM developed 6 and 17 years later, respectively. In most, treatment for MM also improved skin lesions, suggesting that MM treatment should precede standard treatment for IgA pemphigus (15). Indeed, Koga et al., (15) reported daratumumab with lenalidomide plus dexamethasone as an effective therapeutic option for both MM and IgA pemphigus.

HMs less frequently reported as related to true SPD are represented by aplastic anaemia (16), lymphomas (such as CD30+ anaplastic large-cell lymphoma and nodal marginal zone lymphoma) (17) and chronic lymphocytic leukaemia (CLL) (18).

Thorough investigations, with immunofluorescence and whenever possible with immunoblotting, can aid in making the correct diagnosis, differentiating this entity from other immunobullous diseases. It should be kept in mind that, although paraneoplastic pemphigus is commonly associated with HM, HM-associated SPD-type IgA pemphigus is a distinct disease and that the terms should not be used interchangeably (19).

Besides therapy of the underlying HM, which tends to work best also for the associated skin condition, dapsone remains cornerstone in treating SPD and SPD-type IgA pemphigus, starting at a dose of 25 mg, with a target of 50–150 mg/day; the lowest dose necessary to control symptoms should be maintained, and monitoring of hematologic toxicity is mandatory (20). Other therapeutic options include topical and oral corticosteroids (used concurrently or as primary treatment), immunosuppressants such as mycophenolate mofetil and azathioprine, phototherapy, photochemotherapy and acitretin (20). Isolated refractory cases have been managed with tumor necrosis factor (TNF)-α antagonist infliximab and adalimumab, intravenous immunoglobulin or plasmapheresis (10). HM-associated cases, however, are most commonly managed with systemic corticosteroids and dapsone (10–18).

#### 2.1.2 Neutrophilic eccrine hidradenitis (level of evidence 4)

Neutrophilic eccrine hidradenitis (NEH) is a rare, self-limiting ND of unknown aetiology with a characteristic histopathologic pattern, typified by neutrophil-rich infiltrates and necrosis of eccrine sweat glands. The clinical picture is highly heterogenous, manifesting with asymmetric, erythematous-oedematous papules or plaques of variable size, either asymptomatic or pruriginous and painful, closely related to Sweet syndrome (SS) (21). Concordantly, the differential diagnosis of NEH is wide, possibly including SS, erythema multiforme, vasculitis, bacterial (e.g., Pseudomonas) folliculitis and idiopathic eccrine palmoplantar hidradenitis, which is a self-resolving disease observed in childhood.

NEH was initially described in patients with acute myeloid leukaemia (AML) receiving cytarabine (22). Intriguingly, patients with underlying HMs who develop NEH, commonly do so after the first cycle of systemic chemotherapy (23). There is some evidence supporting direct drug toxicity of chemotherapy to the eccrine sweat gland (due to preferential concentration) acting as a trigger for the onset of NEH (24). From a pathophysiologic standpoint, NEH could represent a chemotherapy-induced reactive disorder in the context of an abnormal neutrophil response (25).

Of note, NEH has also been identified in untreated cases of AML and chronic myeloid leukaemia (CML) (22) and, although very rarely, in idiopathic cases (26), the latter being successfully treated with colchicine (26). In such cases, age-appropriate cancer screening has been strongly recommended (21).

Being a self-limiting disease, NEH does not strictly require therapy, but may be managed only with supportive care (27). Moreover, in cases of NEH related to a specific chemotherapeutic agent, dapsone may be useful before drug rechallenge (28).

### 2.2 Dermal neutrophilic dermatoses

#### 2.2.1 Sweet syndrome and related disorders (level of evidence 3A-4)

**Figure 1:**
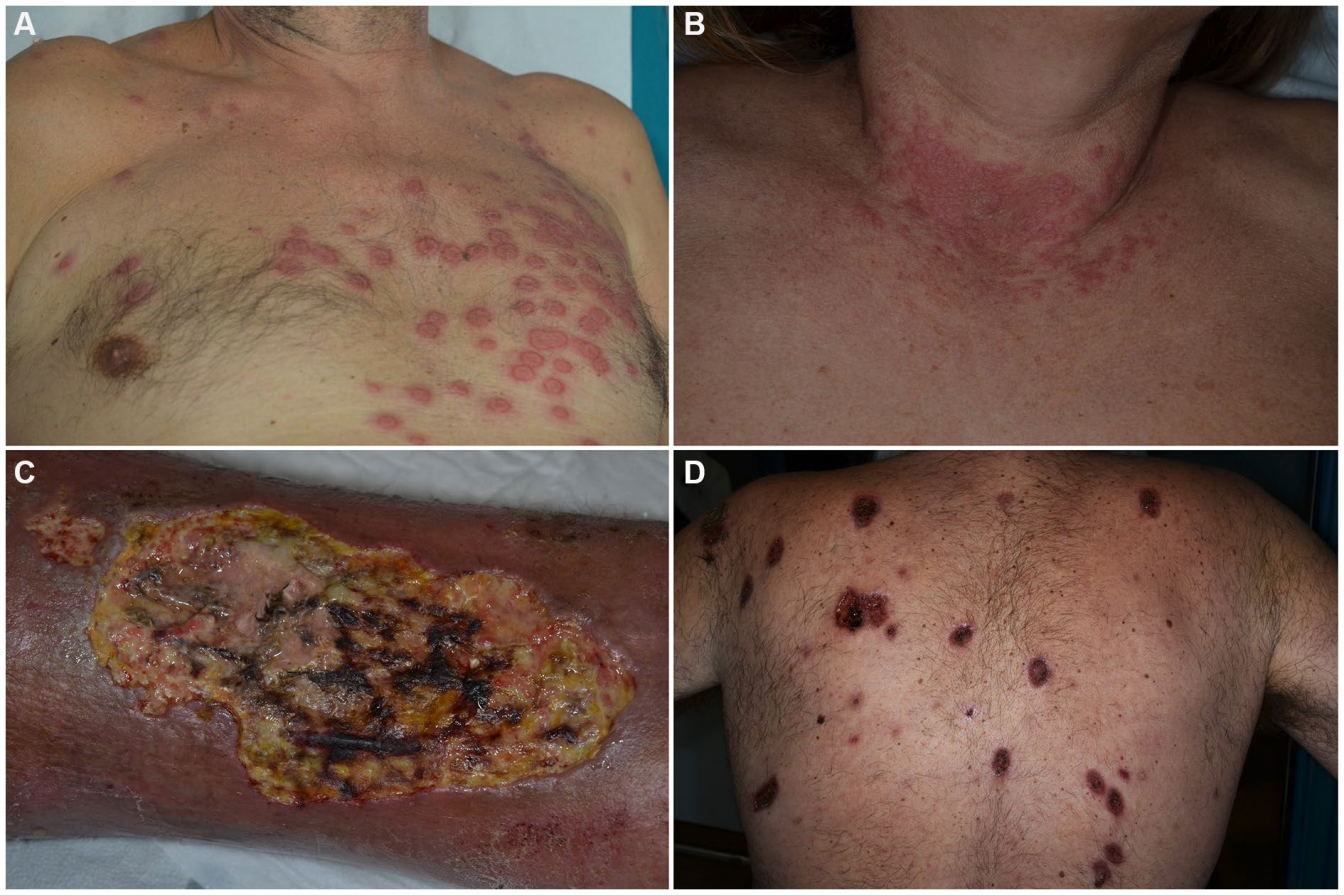
Clinical features of Sweet’s syndrome **(A,B)** and pyoderma gangrenosum **(C,D)**. Erythematous, infiltrated roundish plaques are observed in the prototypic form among dermal neutrophilic dermatoses **(A,B)**. Ulcerations with typical violaceous, undermined borders are seen in pyoderma gangrenosum, either in its unilesional or multilesional presentation **(C,D)**.

SS is a rare ND clinically characterized by the sudden onset of painful, tender, well-demarcated, erythematous papules, plaques and nodules, usually accompanied by fever (>38°C), leukocytosis and elevation of inflammatory markers (Figures 1A,B) (29). Arthralgias, malaise, headache and myalgias may concur. On histology, a dermal infiltrate of mature neutrophils without overt vasculitis is typically observed. Although SS is mostly considered as idiopathic, drug-induced or reactive cases arising in the setting of infectious, inflammatory and neoplastic diseases are recognized. HMs account for 85% of malignancy-associated cases, with AML being the most common associated form. Other entities include myelodysplastic syndromes (MDS), non-Hodgkin lymphomas (NHL), CLL and MM. Cutaneous lesions can present before, after, or simultaneously with malignancies and do not seem to have prognostic implications (30). No clear association has been demonstrated between clinical presentation and the aforementioned settings of occurrence. However, HM-associated SS has been suggested by some to present more frequently in older patients (31), with no sex preference and in those with complete blood count abnormalities (31–33). Also, a lack of arthralgias (31, 33) as well as a more persistent or recurrent course has been described (30).

HM-associated forms have been traditionally linked with a more histiocytoid histological appearance, which is defined by the presence of morphologically immature neutrophils that resemble histiocytic elements. While the link between histiocytoid SS and HMs has been questioned (34), a recent systematic review confirmed the association, especially with recurrent cases of histiocytoid SS (35).

Classic diagnostic criteria for SS include Su and Liu’s set first published in 1986 (36) and then modified by von den Driesch (37) and a separate diagnostic framework for drug-induced SS which was authored by Walker and Cohen (38). The most recent revision by Nofal et al. builds on von den Driesch’s work and distinguishes two constant features (abrupt onset of painful or tender erythematous papules, plaques, or nodules with a dense dermal neutrophilic infiltrate on histology) and a series of variable ones (fever >38°C; atypical skin lesions including hemorrhagic blisters, pustular lesions, cellulitis-like-lesions; presence or absence of leukocytoclastic vasculitis; subcutaneous, histiocytoid, xanthomatoid or cryptococcoid variant on histology; elevated erythrocyte sedimentation rate; elevated C-reactive protein levels; leukocytosis; neutrophilia; anemia), that may help avoid misdiagnosis (39).

**Figure 2:**
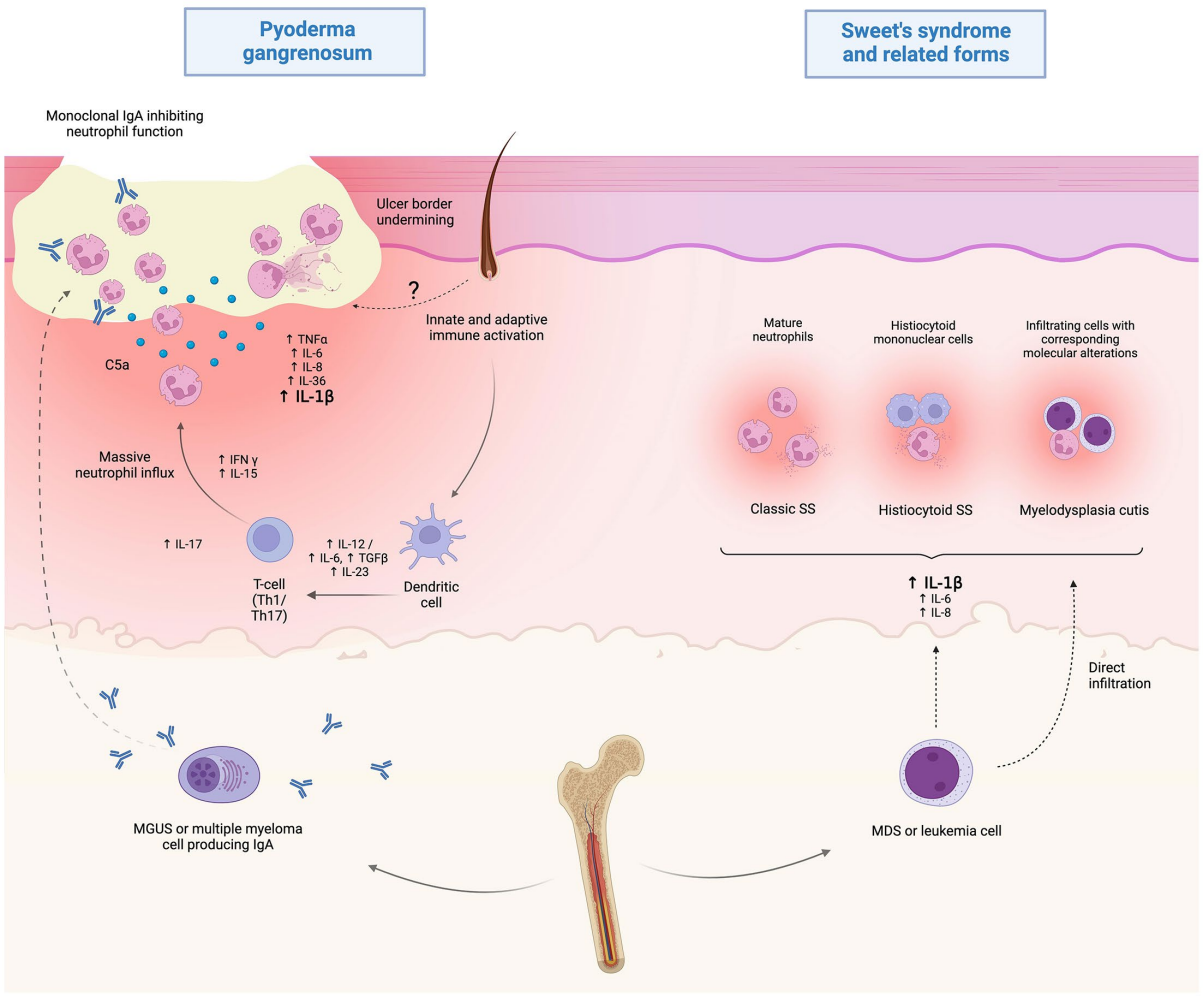
Proposed pathophysiology of hematological malignancy (HM)-associated Sweet’s syndrome (SS) and pyoderma gangrenosum (PG). While the current understanding of HM-associated PG is limited, it is speculated that immunoglobulin A paraproteinemia may play a role by altering neutrophil function in MGUS or multiple myeloma-associated cases. Myelodysplastic or frankly leukemic cells may promote systemic inflammation, resulting in the clinico-pathological picture of SS. Also, direct infiltration of myelodysplastic cells may play a role in the recently disclosed setting of myelodysplasia cutis. Created with BioRender.com.

With specific regard to MDS-associated forms, a spectrum of so-called myeloid dermatoses has been proposed, including: (i) classic clinicopathologic pictures of SS, (ii) histiocytoid SS and (iii) leukemia cutis, which is distinct, is positioned at the more severe end of the spectrum and is typified by true blast infiltrates in the skin. Representing *de facto* leukemic progression in the skin, leukemia cutis portends a poor prognosis and presents with either localized or more frequently widespread erythemato-violaceous papules, nodules or masses (40). Recently, this spectrum has expanded to embrace also an intermediate entity, which has been termed myelodysplasia cutis and may be diagnosed upon documentation of dermal infiltrates composed of non-blast myeloid cells clonally related to MDS cells in the bone marrow (41) (Figure 2).

Clinically, myelodysplasia cutis may present either with features of classic SS, i.e., erythematous, edematous plaques, or with a diffuse papulonodular eruption superimposable to that of leukemia cutis (42). This disconnect highlights the limitations in the current understanding of the pathophysiology of this spectrum and simultaneously underscores the importance of performing thorough investigations in otherwise typical SS cases.

Interestingly, a proportion of SS cases classified as classic or histiocytoid SS (43) as well as myelodysplasia cutis (42) may present in the setting of VEXAS syndrome, a newly recognized, autoinflammatory disease (43).

First described in 2020 in middle-aged men with a series of different, late-onset autoinflammatory manifestations, VEXAS is due to an inactivating acquired mutation in UBA-1, a gene encoding the ubiquitin-activating enzyme E1 (1). Disruption of ubiquitylation leads to altered degradation of proteins and their accumulation as cytoplasmatic vacuoles in an aberrant myeloid precursor, which is thus given a proliferative advantage and determines myeloid-induced inflammation. It is noteworthy that also drug-induced proteasome inhibition leads to SS in some cases (44).

VEXAS syndrome has a high morbidity and may present with fever, cytopenia, vacuoles in myeloid and erythroid precursor cells, bone marrow morphologic dysplasia and cutaneous and/or systemic autoinflammatory manifestations, such as ND (typically SS), vasculitis, relapsing polychondritis and pulmonary sterile neutrophilic infiltrates. It is noteworthy that cutaneous lesions in VEXAS-associated SS may have an arcuate papular morphology in up to a third of cases, which may be a clue to the underlying condition (45, 46). Importantly, it is presently unclear whether VEXAS cases classified as classic SS are true SS or were simply inadequately investigated from a molecular standpoint, lacking demonstration of an identical mutation in both skin and blood.

While dealing with SS in patients with HMs, it should be kept in mind that some cutaneous forms can also be triggered by medications commonly used for the neoplasm itself, like granulocyte colony stimulating factor (G-CSF), all-trans retinoic acid and hypomethylating agents (47).

Concerning possible treatment options, classic SS usually shows an excellent response to systemic steroids, such as prednisone at a dosage of 0.5–1 mg/kg/day, with a slow tapering within 4–8 weeks. Conversely, HM-associated as well as VEXAS-associated SS cases may experience a steroid-refractory, chronic-relapsing course, with the latter representing a possible clue to the underlying condition (30). In such instances, treatment of the neoplastic disorder represents the first choice followed by systemic corticosteroids and/or immunomodulating/immunosuppressive agents. For SS in VEXAS cases, particularly, while methotrexate, cyclosporine, and anti-interleukin (IL)-1 or anti-IL-6 inhibitors tend to result in transient responses, Janus kinase (JAK) inhibitors, e.g., ruxolitinib, are shaping as a more effective approach, leading to dramatic and even durable remissions (48, 49).

Finally, myelodysplasia cutis responds to azacytidine or hypomethylating agents, but hematopoietic stem cell transplantation remains the only curative option for both myelodysplasia cutis and VEXAS (42, 43, 50).

#### 2.2.2 Erythema elevatum diutinum (level of evidence 4)

Erythema elevatum diutinum (EED) is a cutaneous leukocytoclastic vasculitis also classified as a dermal ND, due to its prominent neutrophilic component at histology in the early phases, its peculiar course, and its evolution, that set it apart from other vasculitides (51).

EED initially presents with symmetric, erythemato-violaceus, soft papules and plaques that favor the extensor surfaces of acral body sites and become indurated over time. The clinical evolution reflects the differences in histopathological appearance over time. In the early stages, a dermal infiltrate of polymorphonuclear cells along with fibrin deposition and sometimes SS-like papillary oedema is observed. As the lesions enter the chronic phase, histiocytes become more prevalent, with vascular prominence, lipid deposition, possible spindle cell proliferation and the characteristic finding of progressive concentric perivascular fibrosis (51).

The pathogenesis of EED is still incompletely understood but it is postulated that it originates from chronic antigenic exposure or excess antibody levels. Immune complex deposition occurring in post-capillary venules as a result of infections, hematologic or autoimmune diseases, may then lead to the production of key cytokines, such as IL-8, setting the inflammatory process in motion, similarly to other ND (51).

Several associations have been recorded, including infections, particularly HIV, autoimmune disorders and HMs (52). Among the latter, paraproteinemias (35.3%) hold a prominent role, particularly cases of the IgA-type, but other plasma cell dyscrasias, NHL, MDS, hairy cell leukemia and CLL are also possible (53). Typically, cases last 5–10 years before undergoing self-resolution; however, those associated with IgA paraproteinemia may persist for longer.

Finally, co-occurrence with other ND, such as pyoderma gangrenosum (PG), has also been observed, highlighting the association of HMs with most members of the ND spectrum (54).

### 2.3 Dermal/hypodermal

#### 2.3.1 Pyoderma gangrenosum (level of evidence 3A-4)

PG is an autoinflammatory polygenic skin disorder classified within the group of deep/hypodermal ND and clinically characterized by rapidly evolving cutaneous ulcers, with undermined borders and peripheral erythema (Figures 1C,D) (55). In addition to the classic ulcerative form which accounts for 85% of cases, PG can occur in other variants such as: bullous, pustular, vegetative, peristomal, genital, infantile and extracutaneous, with individual cases sometimes switching between variants (56). Several mimickers of PG have been identified, the most important being venous ulcers, deep infections, vasculitides (especially ANCA-associated ones) and neoplasms, such as primary cutaneous CD30+ anaplastic large cell lymphoma.

Criteria have been developed to aid in the diagnosis of this entity. The first set of criteria was published by Su et al. in 2004 (57). More recently, criteria for the classic ulcerative variant have been validated by means of a Delphi consensus of international experts, emphasizing the role of histology (58). The third set of criteria, the PARACELSUS score, has been proposed by a German group, particularly for the differential diagnosis with venous leg ulcers. It represents a weighted score incorporating several items (3 points for major criteria: Progressive course of disease, Absence of relevant differential diagnoses, Reddish-violaceous wound border; 2 points for minor ones: Amelioration due to immunosuppressant, Characteristically bizarre ulcer shape, Extreme pain >4 VAS, localized pathergy phenomenon; 1 point for additional ones: Suppurative inflammation in histopathology, Undermined wound margin, Associated systemic disease), whereby a sum of 10 or more strongly supports a diagnosis of PG (59). Among the three, the PARACELSUS score correctly identifies the highest proportion of PG patients (60), but research efforts are ongoing to device newer and better diagnostic criteria, also for clinical trials (61).

An association with systemic diseases is documented in 50% of PG cases (22). HMs, particularly, may be present in up to 10% of patients with PG (62), with AML, CML, MM, MDS, and MGUS being the most frequent associated diagnoses (4).

In a large retrospective cohort study conducted by Ashchyan et al. (63), patient age was shown to influence the risk for certain comorbidities; patients aged 65 years or older had a higher probability of having associated HMs than younger patients. However, other studies have since reported concomitant HMs also in younger PG patients (e.g., average age of 56.6 years), highlighting that a hematologic work-up should not be restricted to patients of a certain age (64).

According to a recent systematic review on HM-associated PG (64), MDS (24.4%) and IgA-type MGUS (22.1%) represent the two most frequent associations. As both disorders can progress to overt malignancies, such as AML - reported in 11.5% of cases - and MM, respectively, it is important to identify patients with MGUS or MDS early and monitor them closely for disease progression, so that appropriate treatment can be started promptly (64). Indeed, the same authors pointed out that in most cases the diagnosis of MDS and MGUS preceded the onset of PG, whereas it was made concurrently in patients with AML. Of note, while ulcerative PG accounted for the majority of HM-associated PG, the bullous variant, which is usually characterized by a very severe and rapidly progressing picture, has been recorded in up to half of cases associated with AML (64). From a pathogenetic standpoint, while paraproteinemia-associated cases are speculated to result from an IgA-mediated impairment of neutrophil function and/or altered chemotaxis, little is known about the exact mechanisms underlying MDS-associated forms.

PG management encompasses a variety of systemic, topical and wound care options, which can be chosen and combined based on disease extent, inflammatory versus non inflammatory phase and comorbidities (55). In a proportion of cases, comorbidity-directed therapies aimed to control the associated HM also result in PG remission, proving indirect insights into the disease pathogenesis. Although chemotherapy alone led to healing of HM-associated PG in just 7.5% of reported cases (64), figures may be different with newer treatment approaches for HM.

In different case reports, PG in the setting of MDS has been successfully treated with a combination of systemic corticosteroids, immunosuppressive and immunomodulators agents (64–66).

Thalidomide, particularly, by modulating the release of inflammatory mediators like TNF-α and inhibiting the chemotaxis of monocytes and leucocytes and phagocytosis by neutrophils (67), has been shown to be very effective for both MDS and PG (68–71).

Similarly, MGUS-associated PG cases experienced partial and/or complete remission following MGUS-directed treatments, including autologous peripheral blood stem cell transplantation (72) and, particularly, after the administration of novel oral agents.

In detail, dramatic responses to bortezomib, a proteasome inhibitor, have been reported in PG cases with IgA MGUS (73–75); and smoldering MM (75). It is particularly noteworthy that switch to bortezomib resulted in prompt healing of either giant (74) or anti-TNF-α refractory PG (63) in just 1 month. Similarly, ixazomib, a next generation oral proteasome inhibitor, was reported to be effective in a case of PG with concurrent IgA smoldering MM. Strikingly, a complete response was achieved in just a few weeks since initiation, paralleling the resolution of MGUS (76). Indeed, an aggressive management of HM aimed at the resolution of concomitant IgA MGUS has been advocated for to achieve PG remission (77).

Overall, the development of PG in cases of HMs may confer a worse prognosis to the underlying disease and an appropriate haematological and clinical work-up should be mandatory. Also, careful consideration should be given prior to prescribing cyclosporine and TNF-α inhibitors in patients with HMs associated to PG, to avoid immunosuppression (78).

## 3 Eosinophilic dermatoses

### 3.1 Eosinophilic annular erythema (level of evidence 4)

Eosinophilic annular erythema (EAE) is a rare, superficial eosinophilic dermatosis (ED) that manifests with severely pruritic, large annular plaques hallmarked by intense peripheral erythema and central pigmentation. It was originally described in children (hence the name annular erythema of infancy) but can occur also in adults. Previously classified as a superficial variant of Wells syndrome (WS), EAE is an autonomous entity with a wide range of disease associations. The diagnosis is reached via clinicopathologic correlation, with several clues allowing to differentiate it from WS. Aside from the predominance of annular lesions, in EAE the inflammatory infiltrate is mainly perivascular (not deep dermal as in WS) and no flame figures are observed (at least in typical cases) (79). In a nation-wide multicenter French study, 4/18 (22.22%) patients had a concomitant HM, including polycythemia vera (*n* = 2) and B-cell lymphoma (*n* = 2). However, a lower rate was reported in the literature (80). While the peculiar cutaneous picture of EAE should prompt the consideration of screening for underlying malignancy, especially in the elderly, the strength of the relationship with HMs is subject to debate. Also, some cases of EAE may represent figurate variants of eosinophilic dermatosis of hematologic malignancy (EDHM) (81).

### 3.2 Eosinophilic dermatosis of hematologic malignancy (level of evidence 4)

EDHM represents a chronic-relapsing pruritic disorder occurring primarily in patients suffering from B-cell neoplasms, particularly CLL in which 6–8% of patients may be affected (accounting for up to 77% of EDHM cases), but also NHL (such as mantle cell lymphoma), acute leukemias, MM/MGUS and even T-cell lymphomas (82).

Now classified within the spectrum/group of ED (6, 82–84), the nomenclature of EDHM has undergone several changes since its original description, passing from exaggerated delayed hypersensitivity to mosquito bite in CLL (85) to insect bite-like reaction in patients with HMs (86) to ED of myeloproliferative neoplasms (MPN) (87) to EDHM (88). Two alternative names, i.e., T-cell papulosis associated with B-cell malignancy (89) and hematologic-related malignancy-induced eosinophilic dermatosis (He Remained) (90), have then been proposed, emphasizing its morphological features and the pathophysiological relationship with HMs, respectively (82). Finally, the all-comprehensive definition of polymorphic eruption of HMs has been proposed to embrace the full spectrum of EDHM clinicopathological nuances as well as its follicular variants (4).

**Figure 3:**
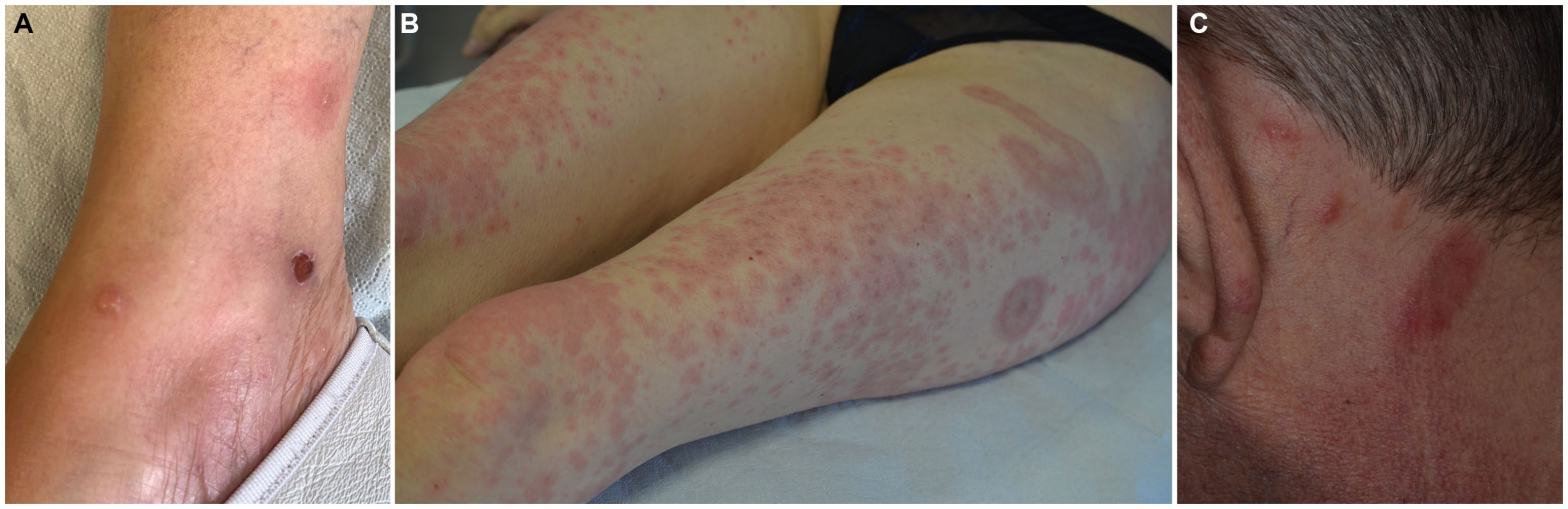
Spectrum of clinical manifestations in eosinophilic dermatosis of hematological malignancy, including pemphigoid-like **(A)**, Wells syndrome-like **(B)** and eosinophilic pustular folliculitis **(C)** presentations.

The typical picture of EDHM consists of recurrent papules, papulo-vesicles, plaques, nodules, urticarial lesions and tense bullae, distributed to the limbs, trunk, and head and neck area in decreasing order of frequency (Figures 3A–C). Exceptionally, eyelid involvement has also been observed (91).

Intense itching is reported, and secondary excoriation is often noted.

It is noteworthy that pemphigoid-like tense bullae have been described in up to a third of cases (84, 92) highlighting the importance of performing immunofluorescence studies. A third, smaller subset of patients presents with eosinophilic cellulitis-like forms (4).

While evidence on seasonal variation is controversial (89, 92–94), it has progressively become apparent that a substantial proportion of cases occurs independently of actual insect bites.

**Figure 4:**
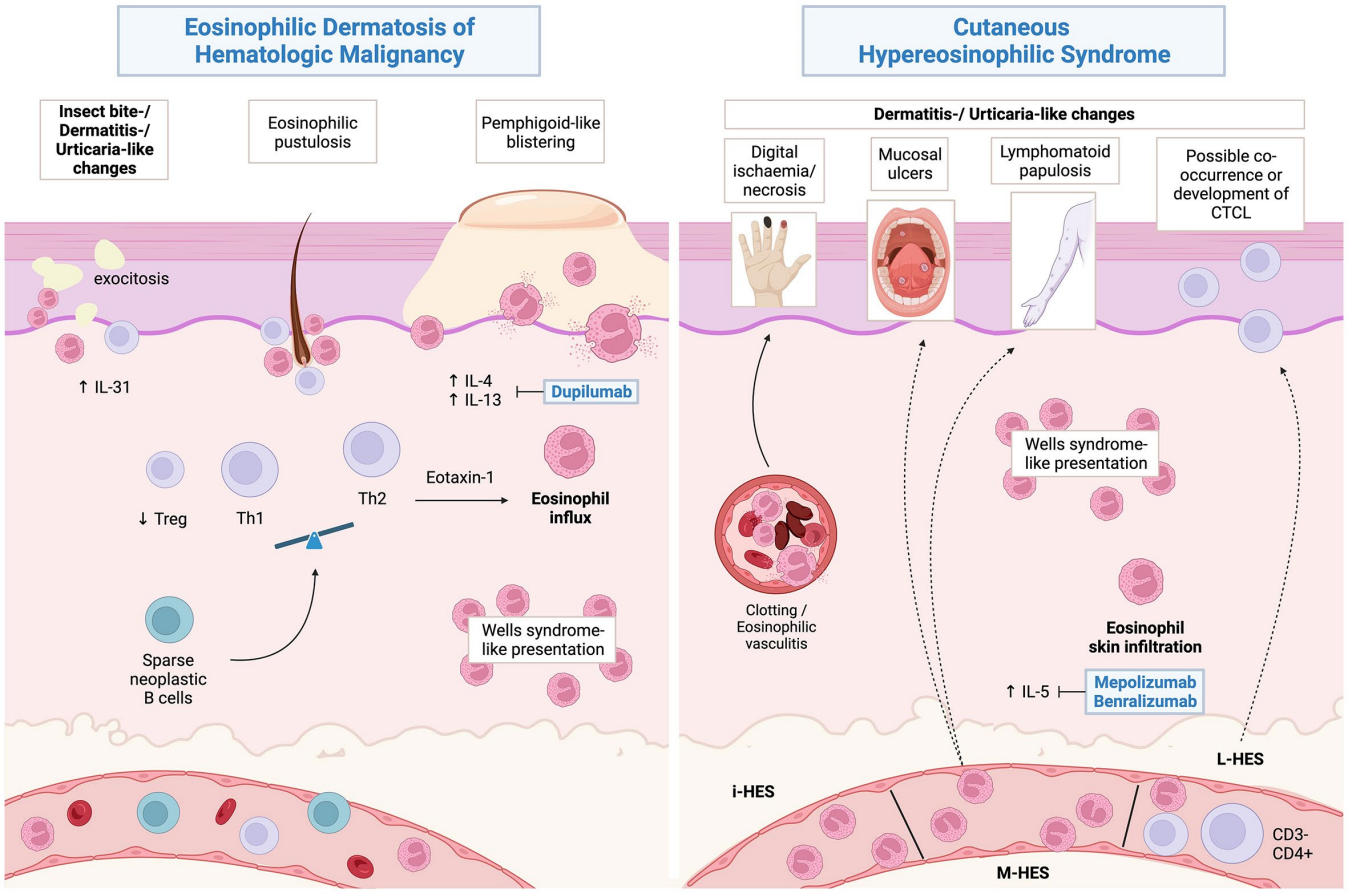
Proposed pathophysiology of eosinophilic dermatosis of hematological malignancy and possible associations of hypereosinophilic syndromes. Created with BioRender.com.

From a pathophysiological perspective, EDHM is T helper 2 (Th2)-skewed process, showing a reduced FOXP3/CD4 ratio (indicating a relative deficiency in the regulatory T -TReg- compartment) and significant IL-4, IL-31 and eotaxin-1 overexpression in the skin. Notably, serum IL-4 is also significantly elevated (84). Skin presence of the same neoplastic B-cell clone as in extracutaneous tissues was demonstrated in 13/15 (86.6%) cases associated with CLL, leading to speculation on a possible direct role in EDHM pathogenesis (89, 95, 96). One unifying hypothesis would have an inconspicuous proportion of neoplastic B-cell clones infiltrating the skin and orchestrating a shift toward type 2 immunity, with subsequent eosinophil chemotaxis (84) (Figure 4). While cases with non-eosinophil-rich histologic pictures have been described (89), it is important to underscore that these can relapse showing typical EDHM histology and *viceversa* (4).

Eosinophilic (pustular) folliculitis and acneiform follicular mucinosis may also be part of the EDHM spectrum, demonstrating superimposable histological features (4, 97). A series focusing on folliculotropic forms reported a higher proportion of head and neck involvement (89). Indeed, a recent study evaluating cutaneous hypersensitivity reactions in a cohort consisting of 501 patients with CLL/small lymphocytic lymphoma (SLL) showed a predominately folliculocentric CD4+ T-cell infiltrate in 5/17 cases, all occurring in the head and neck area (94). In some rare instances, the infiltrates may also involve the subcutaneous tissue, with a clinicopathologic picture of eosinophilic panniculitis (98).

Due to its protean manifestations, EDHM poses a significant challenge in diagnostic terms, with possible differential diagnoses including bullous pemphigoid, true insect-bite reaction, eczema, urticaria, urticarial vasculitis (99), leukemia cutis and disseminated Herpes zoster (100). Mycosis fungoides, especially the follicular variant, is sometimes listed as a possible differential diagnosis of eosinophilic folliculitis/folliculotropic EDHM.

Originally, Byrd et al. proposed a set of criteria to support the diagnosis of EDHM including: (i) pruritic, papular, nodular, and/or vesiculobullous eruption that is resistant to conservative management; (ii) a superficial and deep eosinophil-rich dermal lymphohistiocytic infiltrate on histology; (iii) exclusion of other causes of tissue eosinophilia; and (iv) pre-existing diagnosis of HM (87). However, said criteria fail to incorporate all the nuances in the EDHM spectrum (4).

It should be underscored that EDHM holds no prognostic relevance with respect to the underlying HM (93); however, it may benefit from treatments targeted at the associated HM. In the past, systemic and topical corticosteroids, dapsone, other nonspecific immunosuppressant/immunomodulating agents (e.g., doxycycline) as well as UVA1 light phototherapy have proved effective at controlling the cutaneous picture (92, 93). While the condition is responsive to a variety of options, relapses are frequent (92) and long-term maintenance may be required. Recently, dupilumab an anti-IL4Rα monoclonal antibody (mAb) targeting type 2 inflammation demonstrated high effectiveness in a good proportion of EDHM cases (5/9) (101–104), paving the way for pathogenesis-driven therapy of this challenging condition.

### 3.3 Wells syndrome (level of evidence 3A-4)

WS is a dermal eosinophilic dermatosis, mainly observed in adults and hallmarked by a benign, yet sometimes recurrent clinical course (105). WS classically presents with urticarial erythematous-edematous plaques, but sometimes also with more infiltrated lesions, vesicles, or blisters. An annular configuration with central blanching and a purplish border may be observed. Generally, complete remission of a flare occurs within 4 to 8 weeks from onset, but a more prolonged course is possible, up to several years (105, 106).

Many clinical varieties of WS have been identified, including plaque-type (most common in children), annular granuloma–like (most commonly seen in adults), urticaria-like, papulovesicular, bullous, papulonodular and fixed drug eruption–like (106).

From a histopathological point of view, three stages are described. At the first stage of the disease, only dermal edema along with eosinophilic infiltrates is documented. A second (sub-acute) phase then ensues, being characterized by the presence in the mid-deep dermis of the so-called “flame-figures,” i.e., structures made of degenerated collagen fibers and eosinophils at the center with a dense infiltrate of histiocytes admixed with numerous eosinophils at the periphery. With time, as lesions enter the third phase, only granulomatous changes with histiocytes and giant cells around the “flame figures” are seen. Vasculitic changes are not observed in WS. It is important to underscore that while characteristic, “flame-figures” are not pathognomonic of WS, possibly appearing also in other forms of the eosinophilic dermatosis spectrum (105).

Two sets of diagnostic criteria have been proposed by Caputo et al. and Heelan et al., in 2006 and 2013, respectively, (106, 107). The most recent one requires two out of four major criteria (documentation of any of the previously reported clinical variants; relapsing, remitting course; no evidence of systemic disease; eosinophilic infiltrates with no vasculitis on histology) alongside at least one out of four minor criteria (flame figures; granulomatous changes on histology; peripheral eosinophilia not persistent and not greater than >1,500/μl; presence of a triggering factor) (107).

Whether distinguishing Wells syndrome (WS) from EDHM or cutaneous hypereosinophilic syndrome (cHES) (108) in hematologic patients is possible is still matter of debate (109).

Indeed, a proportion of cases originally reported as WS in patients with B-cell malignancies (mantle cell lymphoma, CLL) may have been clinically consistent with EDHM (110–112). Vice versa, clinically typical WS, with large erythematous, oedematous areas, has been recognized in some cases of CLL (113, 114). Co-occurrence of WS with EDHM in patients with B-cell neoplasms credits the idea of one spectrum of disorders, with different nuances in clinical expressivity (113, 115, 116).

Eosinophilic cellulitis may also be a presenting feature of idiopathic HES (108, 117, 118), rarely showing a more severe, necrotic evolution (119).

Recurrent eosinophilic cellulitis has been reported - albeit anecdotally - also in myeloid/lymphoid neoplasms with eosinophilia harbouring the FIP1L1-PDGFRA fusion transcript (120) or t(5,12)(q33;p13) translocation (121).

It is important to underline that WS-like cutaneous features may be present both in patients with EDHM and in those with HES, peripheral blood eosinophilia being the major distinguishing feature between the two scenarios.

Classic treatment options for WS include topical and systemic corticosteroids tapered over the course of several weeks, as well as cyclosporine or dapsone either as steroid-sparing agents or as add-on for refractory cases (122). Interestingly, newer drugs capable of selectively targeting type 2 immunity, such as anti-IL-4/13 (123, 124), anti-IL-5 (125, 126), anti-IL-5R (127) and JAK2 inhibitors (128) have been reported to be effective in isolated cases of WS, similarly to both EDHM and HES. Dapsone and biologics are of particular interest as they appear to control cutaneous manifestations without acting as immunosuppressants.

Cases of WS associated with HM have been traditionally managed with systemic corticosteroids, usually with rapid responses (113, 115, 116).

### 3.4 Hypereosinophilic syndrome and cutaneous hypereosinophilic syndrome (level of evidence 3A-4)

HES is a condition defined by the presence of peripheral blood hypereosinophilia (≥1.5 × 10^9^/L) in association with tissue/organ damage. According to the recent international consensus classification (129), the following etiologic scenarios are recognized: (i) secondary/reactive HES (eosinophils are reactive and non-clonal) including lymphocyte variant-HES (L-HES); (ii) primary HES (associated with a hematopoietic neoplasms); (iii) idiopathic HES. The hematologic neoplasms associated with the second scenario include: myeloid/lymphoid neoplasms with eosinophilia and tyrosine kinase gene fusions (M/LN-eo-TK); eosinophilia associated with other myeloid neoplasms, e.g., CML or AML with inv. (16); and chronic eosinophilic leukemia, not otherwise specified (CEL, NOS). In contrast, the term of “idiopathic HE” or “HE of unknown significance” (HEus) is used to describe persistent hypereosinophilia (≥6 months) without associated organ/tissue damage (129).

Concerning skin manifestations of HES, while a typical picture consisting of eczema- or urticaria-like features can broadly be defined, some nuances are characteristic of each form and will be discussed separately. Hopefully, a better categorization of cHES is to be expected as our molecular understanding of HES becomes progressively more detailed.

Lymphocyte-variant HES is linked to clonal circulating Th2 CD4 T cells (>0.5% T cells), most commonly with a defective CD3− CD4+ immunophenotype and shows the greatest frequency (79%) of cutaneous involvement among HES forms (130). In line with its Th2 polarization, L-HES commonly presents urticaria/angioedema, pruritus and eczematous lesions. Unusual manifestations include subcutaneous nodules and palmoplantar haemorrhagic blisters (131). Of note, although a certain degree of overlap exists between EDHM and cHES (e.g., nonspecific erythematous eruptions, pruritus, urticaria, eosinophilic cellulitis), true, pemphigoid-like blistering is only rarely observed in HES (131).

As both nodal and primary cutaneous peripheral T-cell lymphomas may develop during the course of L-HES, close follow-up may be advised. Of note, cHES may also initially masque a concomitant cutaneous lymphoma. In a large series, where skin T-cell clonality was available for eight cases, a dominant clone identical to the blood T-cell clone was found in seven, of whom two had cHES alone and five had concurrent primary cutaneous T-cell lymphoma (132).

Primary HES, formerly identified as myeloid HES comprising also CEL, NOS, affects the skin in 23.1–25% of cases (130), with more robust data available for the predominant subgroup harbouring the FIP1L1–PDGFRA fusion gene (32%) which results in a constitutively activated platelet-derived growth factor receptor-alpha (PDGFRA) (133).

Besides the rare occurrence of eosinophilic cellulitis, M/LN-eo-TK with the FIP1L1–PDGFRA fusion gene usually presents with transient erythematous (eczema-like) eruptions (121), pruritus, urticaria and dermographism (130).

Lymphomatoid papulosis, cutaneous nodules, purpura and mouth ulcers have been reported as well (133, 134). Indeed, chronic recurrent ulcerations affecting the oral or genital mucosae are a distinctive, incapacitating manifestation of HES and may represent the first sign of the condition (135–138). Mucosal ulcers have been linked particularly with the FIP1L1–PDGFRA fusion gene-positive form (137, 139, 140), occurring in 8/151 of these patients in a large series (133); however, they have been reported also in idiopathic HES (141, 142) and albeit anecdotally in L-HES (143).

Lymphomatoid papulosis is another characteristic association of M/LN-eo-TK with the FIP1L1–PDGFRA fusion gene, co-occurring in approximately 7% (11/151) of cases (133, 144).

Recently, Kitayama et al. demonstrated direct skin infiltration by neoplastic, FIP1L1–PDGFRA fusion gene-positive eosinophils in a patient with M/LN-eo-TK harbouring the mentioned translocation and presenting with a pruritic erythematous maculo-papular rash, dermographism and slight hyperpigmentation (145). The authors also reported an increase in dermal mast cells (145), which may be in keeping with pre-clinical evidence showing synergism between FIP1L1–PDGFRA fusion gene and the Stem Cell Factor/c-Kit pathway, thus promoting mast cell activation and survival (146).

Of note, a complex relationship links the abovementioned MPN and systemic mastocytosis (SM), one that may have had diagnostic repercussions on a proportion of previous reports (145, 147). From a hematologic perspective, it is imperative to differentiate M/LN-eo-TK presenting with a mast cell proliferation (no KIT D816V mutation) from SM with or without an associated myeloid neoplasm (129, 148). Conversely, from a dermatological perspective, the presence of an abundant mast cell component in the skin may hypothetically explain some features of the cutaneous picture such as dermographism and residual hyperpigmentation (145), the latter possibly serving as a clue to it.

Cutaneous involvement in idiopathic HES occurs in approximately a third of cases (130). According to a review on 32 individual patients, it presents as a pruritic and sometimes painful, erythematous-oedematous, papular eruption mainly on the extremities but also on the trunk. Other possible yet nonspecific skin changes include urticaria/angioedema, telangiectasia, palmar erythema or lichenification, cutaneous atrophy, superficial venous thrombophlebitis, hyperpigmentation (149), erythroderma (150) and eosinophilic cellulitis (117, 119).

A peculiar and possibly underrecognized subset of patients with idiopathic HES presents with necrotizing eosinophilic vasculitis, either as single organ vasculitis or with multisystem involvement. Similarly to idiopathic HES, the diagnosis of HES-associated vasculitis requires the exclusion of secondary causes, particularly eosinophilic granulomatosis with polyangiitis (EGPA) (151). HES-associated vasculitis, also known as idiopathic eosinophilic vasculitis, affects the skin in approximately half of cases, manifesting clinically with a picture of pruritic papular or frankly urticarial lesions, followed - in decreasing order of frequency - by purpuric papules, livedo, angioedema and even skin ulcers or digital necrosis (including splinter haemorrhages and nail fold infarcts), sometimes preceded by Raynaud’s phenomenon (151–159). Lesions predominate on the extremities, but the trunk and the head and neck area may also be affected, such as in patients with temporal arteritis (151, 160).

From a practical perspective, the demonstration of a clinicopathologic picture of eosinophilic vasculitis, including the characteristic digital ulcers, may hypothetically favor the diagnosis of idiopathic HES over L-HES or forms associated with myeloid/lymphoid neoplasia (161).

Importantly, cHES with non-eosinophil rich infiltrates on histology has also been observed. Among such rare instances, cases of interstitial granulomatous dermatitis are of particular interest (133, 162, 163). Indeed, the presence of a granulomatous infiltrate in the skin manifesting clinically with roundish erythematous plaques showing characteristic central umbilication and xanthomization may be a distinctive feature of the newly defined M/LN-eo-TK with t(9,12)(q22;p13) ETV6::SYK (164–166).

Treatment of HES is challenging and variant-specific approaches are needed to obtain optimal results.

While corticosteroids represent a first-line option across HES variants, primary forms associated with a HM tend to respond inadequately. Among the latter, presence of specific fusion transcripts, i.e., FIP1L1–PDGFRA, serves as predictor a good clinical and hematologic response to imatinib 100–400 mg die (167). M/LN-eo-TK with other fusion genes or CEL, NOS may also benefit from the same approach (usually requiring higher dosages) or from next generation tyrosine kinase inhibitors; however, treatment-tailoring based on the underlying neoplasm, possibly with stem-cell transplantation, is required to achieve blood and skin remission (129, 130). L-HES is less responsive than the idiopathic variant to systemic corticosteroids and may require additional lines of treatment. Among the latter, pegylated interferon alpha 2a is regarded an effective and well-tolerated option, opposing the Th2 polarization of the condition (168). Regarding biologics, mepolizumab, an anti-IL-5 mAb recently approved for HES without an identifiable secondary non hematologic cause, resulted in good control of most HES manifestations; however, reported skin outcomes are conflicting (169).

In a phase II trial, patients treated with benralizumab, an anti-IL-5R mAb, also demonstrated good clinical and hematological responses. However, among patients with complete individual descriptive data, only 2/4 with L-HES had sustained cutaneous response while the other half showed quick loss of response; those with idiopathic HES generally had substantial (2/3) or at least partial (1/3) improvements of their respective cutaneous pictures (170). This is consistent with two subsequent reports on idiopathic HES (171, 172). Isolated observations also support the effectiveness of dupilumab in idiopathic HES, however larger studies are needed to define its placing, if any (173, 174).

## 4 Conclusion

Neutrophilic and eosinophilic dermatoses associated with HM represent a heterogeneous group of skin conditions, which may either parallel the course of the underlying hematological disorder (PG, SS) or be independent from it (e.g., EDHM). Several entities belonging to both neutrophilic and eosinophilic dermatoses have recently been subject to provisional reclassification, thanks to a better overall understanding of their molecular aspects. EDHM now incorporates also eosinophilic pustular folliculitis as well as pemphigoid-like and Wells-like presentations. Conversely, several different scenarios have been distinguished in addition to classic SS, i.e., histiocytoid SS, VEXAS-related SS and the newly defined setting of myelodysplasia cutis. Better definition of the molecular characteristics of these dermatoses has also led to promising premises for pathogenesis-driven treatments, as for dupilumab in EDHM; however, mechanisms linking each HM to specific cutaneous phenotypes are still incompletely understood and warrant further research.

## Author contributions

CMa: Supervision, Writing – review & editing, Conceptualization, Investigation, Writing – original draft. FD: Investigation, Writing – original draft, Writing – review & editing. CMo: Investigation, Writing – original draft, Writing – review & editing, Supervision. DC: Writing – review & editing. AI: Writing – review & editing. AM: Writing – review & editing, Supervision, Validation.
